# Players’ performance during worst-case scenarios in professional soccer matches: a systematic review

**DOI:** 10.5114/biolsport.2022.107022

**Published:** 2021-08-30

**Authors:** Markel Rico-González, Rafael Oliveira, Luiz H. Palucci Vieira, José Pino-Ortega, Filipe Manuel Clemente

**Affiliations:** 1 Department of Physical Education and Sport, University of the Basque Country, UPV EHU. Lasarte 71, 01007 Vitoria-Gasteiz, Spain; 2 BIOVETMED & SPORTSCI Research group. University of Murcia, San Javier. España; 3 Sports Science School of Rio Maior, Polytechnic Institute of Santarém, 2140-413 Rio Maior, Portugal; 4 Life Quality Research Centre, 2140-413 Rio Maior, Portugal; 5 Research Centre in Sport Sciences, Health Sciences and Human Development, 5001-801 Vila Real, Portugal; 6 MOVI-LAB Human Movement Research Laboratory, School of Sciences, Graduate Program in Movement Sciences, Physical Education Dept., UNESP São Paulo State University, Bauru, Brazil; 7 Faculty of Sports Sciences. University of Murcia, San Javier. Spain; 8 Escola Superior Desporto e Lazer, Instituto Politécnico de Viana do Castelo, Rua Escola Industrial e Comercial de Nun’Álvares, 4900-347 Viana do Castelo, Portugal; 9 Instituto de Telecomunicações, Delegação da Covilhã, Lisboa 1049-001, Portugal

**Keywords:** Football, Peak performance, High intensity, Elite, Game analysis

## Abstract

Since the analysis of worst-case scenarios (WCS) has been increasing knowledge about match demands and possible impacts for the training process, it seems important to summarize the evidence to provide useful information for the soccer community. Thus, the purpose of this systematic review was to summarize the evidence about WCS in professional soccer. A systematic review of PubMed, SPORTDiscus, and FECYT (Web of Sciences, CCC, DIIDW, KJD, MEDLINE, RSCI, and SCIELO) was performed according to the guidelines for performing systematic reviews in sport science. From the 85 studies initially identified, 12 were fully reviewed, and their outcome measures were extracted and analyzed. There was an inverse relationship between the duration of WCS windows and running output during match play. Occurrences of WCS during soccer matches were also position-dependent across studies, at least, when analyzing performance with the total distance covered variable, although different outputs were identified between women and men players. Future research should consider analyzing the impact of contextual variables (match status, team formation, and match location) on peak match values and the weight of these moderators.

## INTRODUCTION

Match running performance in soccer is a well-researched topic in match analysis [1]. Using different tracking approaches, the collection and description of distances covered at different speed thresholds, accelerations, decelerations, or mechanical load parameters have been described considering different situational factors [2], sexes [3, 4], or other variables. The understanding of the typical values of activity profiles provided a better understanding of the volume of running and identification of the weight of each type of running intensity in the volume of running and the consequences for training [5].

Despite the importance of describing the volume of running performed, the relativization of game pace and value (activity profile per minute) is important to consider the players who have not participated in the whole match [6]. Additionally, the relative meters or number of actions performed per minute can also help to identify the typical pace for adjusting some training drills based on running [7]. However, considering the activity per minute includes the whole match and does not take the natural intermittence of the game into account [8]. In fact, due to the variability within the match, it is expected that some moments can be more intense than others depending on what contextual factors are involved in a certain situation [9].

Therefore, the inspection of peak match running demands (also known as worst-case scenarios (WCS)), defined as the most intense period of a match [10], has been progressively growing, in which the standardized distances or actions made per minute are considering for specific time window to extract values of WCS) [11]. Not only can the epoch used play an important role in determining the peak match demands for the distance covered at each running intensity, acceleration, or deceleration, but also the use of different methodological approaches, such as using fixed epochs or rolling averages may significantly contribute to the different values obtained [12].

Naturally, the peak match running demands in shorter epochs are much more intense than in longer epochs independently of sex [13, 14]. Similarly to considering the whole match, the WCS seem also to be dependent on situational factors [10, 15]. Among these, average match demands for the whole match or WCS are highly position-dependent, thus playing positions should be considered since the values significantly differ from position to position [10]. This should be carefully considered for recovery strategies, training interventions and individualization of the training process [16].

Since the analysis of WCS has been increasing knowledge about match load demands and possible impacts for the training process, it seems important to summarize the evidence to provide useful information for the sports community. Despite the publication of a systematic review about the use of microtechnology to quantify the peak match demands in football codes [17], a systematic review is still needed that allows the comparison of peak match demands among time windows, playing positions, sexes and contextual factors in professional soccer. This information will help towards an understanding of the current state-of-the-art and indicate new routes for research into WCS in soccer. Therefore, the purpose of this systematic review was to summarize the evidence about WCS in professional soccer.

## MATERIALS AND METHODS

The systematic review strategy was conducted according to the guideline for performing systematic reviews in sport science [18].

### Search strategy

PubMed, SPORTDiscus, and FECYT, which contains seven databases (i.e. Web of Sciences, CCC, DIIDW, KJD, MEDLINE, RSCI, and SCIELO), were searched for relevant publications prior to March 22, 2021. Keywords and synonyms were entered in various combinations in the title, abstract or keywords: (soccer OR football) AND (“worst case scenario*” OR “most demanding passage*”). Additionally, the reference lists of the studies retrieved were manually searched to identify potentially eligible studies not captured by the electronic searches. Finally, an external expert has been contacted in order to verify the final list of references included in this scoping review in order to understand if there was any study that was not detected through our research. Possible errata were searched for each included study.

### Study selection

A data was prepared in Microsoft Excel sheet (Microsoft Corporation, Readmon, WA, USA) in accordance with the Cochrane Consumers and Communication Review Group’s data extraction template [19]. The Excel sheet was used to assess inclusion requirements and subsequently tested for all selected studies. The process was independently conducted by the two authors (MRG and JPO). Any disagreement regarding study eligibility was resolved in a discussion.

The screening of the title, abstract and reference list of each study to locate potentially relevant studies was independently performed by the two authors. Additionally, they reviewed the full version of the included papers in detail to identify articles that met the selection criteria. An additional search within the reference lists of the included records was conducted to retrieve additional relevant studies. Possible errata for the included articles were considered.

The inclusion and exclusion criteria can be found in table 1.

**TABLE 1:** Inclusion/exclusion criteria.

Item	Inclusion Criteria	Exclusion Criteria
Population	Studies developed with professional soccer players	Studies developed with players from other team sports (basketball, rugby, Australian football, American football, futsal, etc.) or sport.
Intervention	The data were recorded during soccer matches.	The data were recorded during training sessions.
Comparator	–	–
Outcome	Physical/physiological, technical and/or tactical performance outcomes.	Variables from other nature (e.g. psychological).
Study Design	Soccer players performance during the most demanding passages.	–
Additional criteria	Only original and full-text studies written in English	Written in other language than English. Other article types than original (e.g., reviews, conference abstracts, etc.).

### Data Extraction

The following information was extracted from the included original articles: EPTS, level (league) and players´ mean age, sample, playing positions, variable and time epoch.

### Methodological Assessment

Methodological assessment process was performed by two authors (MRG and JPO) using an adapted version of the STROBE assessment criteria for cross-sectional studies [20], looking studies eligible for inclusion. Each article was assessed based on 10 specific criteria (Table 2). Any disagreement was discussed and solved by consensus decision. Each item was evaluated using numerical characterization (1 = completed; and, 2 = non-completed), where a study with 7 points scored was qualified as a low quality (high risk of bias) and a study with 8 points scored was qualified as a study with low risk of bias [20].

**TABLE 2:** Methodological assessment of the included studies.

Reference	1	2	3	4	5	6	7	8	9	10	Quality
Baptista et al. [21]	1	1	1	1	1	0	1	1	1	1	High
Casamichana et al. [22]	1	0	1	1	1	1	1	1	0	1	High
Fereday et al. [12]	1	0	1	1	1	1	1	0	0	1	Low
Martín-García et al. [23]	1	0	1	1	1	1	1	1	0	1	High
Martín-García et al. [24]	1	0	1	1	1	1	1	1	0	1	High
Muñiz-González et al. [25]	1	0	0	1	1	1	1	1	0	0	Low
Oliva-Lozano et al. [13]	1	0	1	1	1	1	1	1	1	1	High
Oliva-Lozano et al. [26]	1	0	1	1	1	1	1	1	1	1	High
Oliva-Lozano et al. [10]	1	0	1	1	1	1	1	1	1	1	High
Riboli et al. [27]	1	0	1	1	1	1	1	1	1	0	High
Riboli et al. [15]	1	0	1	1	1	1	1	1	1	0	High
Trewin et al. [28]	1	0	1	1	1	1	1	1	1	1	High

**Note:** provide in the abstract an informative and balanced summary of what was done and what was found (item 1); state specific objectives, including any prespecified hypotheses (item 2); Give the eligibility criteria, and the sources and methods of selection of participants (item 3); for each variable of interest, give sources of data and details of methods of assessment (measurement). Describe comparability of assessment methods if there is more than one group (item 4); explain how quantitative variables were handled in the analyses. If applicable, describe which groupings were chosen and why (item 5); give characteristics of study participants (item 6); summarize key results with reference to study objectives (item 7); discuss limitations of the study, considering sources of potential bias or imprecision. Discuss both direction and magnitude of any potential bias (item 8); give a cautious overall interpretation of results considering objectives, limitations, multiplicity of analyses, results from similar studies, and other relevant evidence (item 9); give the source of funding and the role of the funders for the present study and, if applicable, for the original study on which the present article is based (item 10).

## RESULTS

### Study identification and selection

**FIG. 1:**
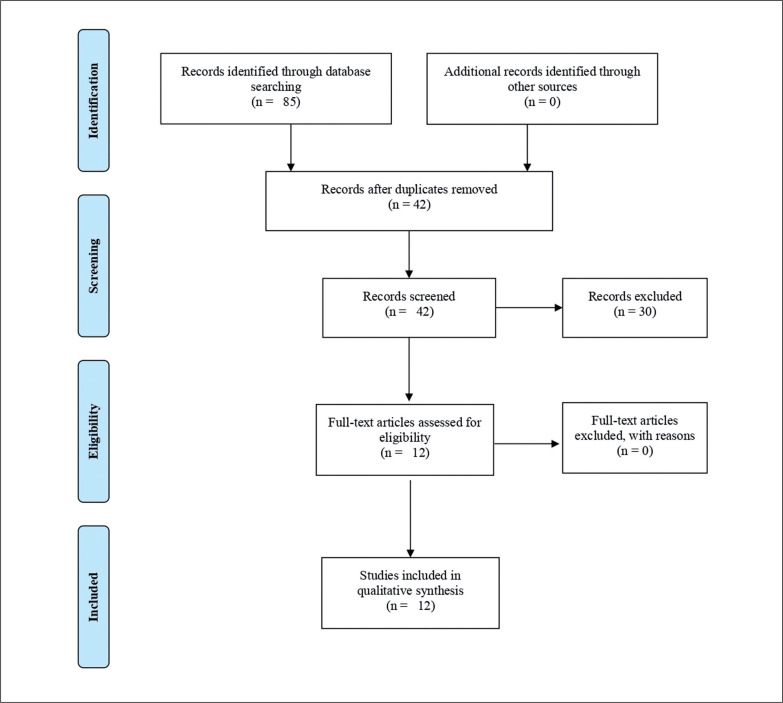
Flow diagram of the selection of studies.

The searching of databases identified a total of 85 titles. These studies were then exported to reference manager software. Duplicates (43 references) were subsequently removed manually. The remaining 42 articles were screened for their relevance based on titles and abstracts, resulting in the removal of a further 30 studies. Following the screening procedure, 12 articles were selected for in depth reading and analysis. After reading full texts, all of these studies were included in the qualitative synthesis (Figure 1).

### Methodological quality

The overall methodological quality of the studies can be found in Table 2.

### Characteristics of individual studies

The characteristics of the included studies are shown in Table 3 (male players: *n* = 10 studies) and 4 (female players: *n* = 2 studies). All studies were developed with professional athletes. Playing position data were included in both tables 3 and 4 with exception for one study [26]. There was one study that data was non-extractable [27]. Both tables 3 and 4 included data by epochs (from 1 min to 10 min) for all variables analysed by each study, respectively.

**TABLE 3:** Performance indicators during most demanding passages in professional male players.

Ref.	EPTS	Level (League) Age	N		Half	Playing position	Variable	Epochs (values per minutes)									
			P	M				1	2	3	4	5	6	7	8	9	10
Baptista et al. [21]	Radio-based tracking system (ZXY Sport Tracking System, Trondheim, Norway)	PRO (Norwegian)	18	15	W	Central defender	Acc (n/min)					1 ± 0					
							Dec (n/min)					1 ± 0					
							HSR (m/min)					15 ± 1					
							Sprint (m/min)					7 ± 1					

						Wide defender	Acc (n/min)					2 ± 0.2					
							Dec (n/min)					2 ± 1					
							HSR (m/min)					24 ± 2					
							Sprint (m/min)					11 ± 1					

						Central midfielder	Acc (n/min)					1 ± 0					
							Dec (n/min)					1 ± 0					
							HSR (m/min)					17 ± 1					
							Sprint (m/min)					6 ± 1					

						Central Forward	Acc (n/min)					2 ± 0					
							Dec (n/min)					1 ± 0					
							HSR (m/min)					21 ± 2					
							Sprint (m/min)					8 ± 1					

						Whole team (Average)	Acc (n/min)					2 ± 0					
							Dec (n/min)					1 ± 0					
							HSR (m/min)					19 ± 2					
							Sprint (m/min)					8 ± 1					

Casamichana et al. [22]	GPS (Viper Pod, 50 g, 88 x 33 mm, Statsports Viper, Northern Ireland)	PRO (Spanish) 21 ± 2	23	37	1st	Central defender	Total distance (m/min)	177 ± 14	142 ± 8	131 ± 8							121 ± 7
							HMLD (m/min)	62 ± 12	36 ± 7	28 ± 4							23 ± 3
							AMP (W·kg-1)	17 ± 1	13 ± 1	12 ± 1							11 ± 1

						Wide defender	Total distance (m/min)	189 ± 16	149 ± 11	137 ± 9							127 ± 7
							HMLD (m/min)	73 ± 16	42 ± 7	35 ± 6							29 ± 5
							AMP (W·kg-1)	19 ± 2	14 ± 1	13 ± 1							12 ± 1

						Midfielder	Total distance (m/min)	196 ± 22	155 ± 17	145 ± 15							135 ± 15
							HMLD (m/min)	70 ± 15	39 ± 8	32 ± 7							27 ± 6
							AMP (W·kg-1)	19 ± 2	15 ± 1	14 ± 1							13 ± 1

						Off. Midfielder	Total distance (m/min)	195 ± 26	157 ± 13	146 ± 12							137 ± 12
							HMLD (m/min)	74 ± 15	45 ± 9	37 ± 8							31 ± 7
							AMP (W·kg-1)	19 ± 2	15 ± 1	14 ± 1							13 ± 1

						Forward	Total distance (m/min)	175 ± 23	136 ± 16	125 ± 14							116 ± 14
							HMLD (m/min)	67 ± 17	39 ± 8	32 ± 7							26 ± 6
							AMP (W·kg-1)	17 ± 2	13 ± 2	12 ± 1							11 ± 1

						Whole team (Average)	Total distance (m/min)	186 ± 22	147 ± 16	136 ± 11							126 ± 14
							HMLD (m/min)	70 ± 16	40 ± 8	33 ± 7							27 ± 5
							AMP (W·kg-1)	18 ± 1	14 ± 1	13 ± 1							12 ± 1

					2nd	Central defender	Total distance (m/min)	176 ± 12	135 ± 9	124 ± 8							114 ± 7
							HMLD (m/min)	64 ± 12	34 ± 6	27 ± 4							22 ± 3
							AMP (W·kg-1)	17 ± 2	13 ± 1	12 ± 1							10 ± 1

						Wide defender	Total distance (m/min)	185 ± 21	143 ± 10	132 ± 9							119 ± 8
							HMLD (m/min)	73 ± 16	40 ± 8	33 ± 5							27 ± 5
							AMP (W·kg-1)	18 ± 2	14 ± 1	13 ± 1							11 ± 1

						Midfielder	Total distance (m/min)	196 ± 22	155 ± 17	145 ± 15							135 ± 15
							HMLD (m/min)	66 ± 15	37 ± 8	31 ± 6							25 ± 5
							AMP (W·kg-1)	18 ± 2	14 ± 1	13 ± 1							12 ± 1

						Off. Midfielder	Total distance (m/min)	192 ± 23	151 ± 13	140 ± 12							127 ± 11
							HMLD (m/min)	73 ± 13	42 ± 7	35 ± 7							28 ± 5
							AMP (W·kg-1)	19 ± 2	14 ± 1	13 ± 1							12 ± 1

						Forward	Total distance (m/min)	192 ± 23	151 ± 13	140 ± 12							127 ± 11
							HMLD (m/min)	64 ± 15	36 ± 9	30 ± 7							24 ± 6
							AMP (W·kg-1)	17 ± 2	13 ± 2	11 ± 1							10 ± 1

						Average	Total distance (m/min)	171 ± 25	131 ± 17	122 ± 14							110 ± 13
							HMLD (m/min)	68 ± 15	37 ± 8	31 ± 6							25 ± 5
							AMP (W·kg-1)	18 ± 2	13 ± 1	12 ± 1							11 ± 1

					W	Central defender	Total distance (m/min)	177 ± 13		139 ± 9		128 ± 8					118 ± 7
							HMLD (m/min)	63 ± 12		35 ± 7		28 ± 4					23 ± 3
							AMP (W·kg-1)	17 ± 2		13 ± 1		12 ± 1					11 ± 1

						Wide defender	Total distance (m/min)	374 ± 19		135 ± 9		135 ± 9					123 ± 8
							HMLD (m/min)	73 ± 12		41 ± 8		34 ± 6					28 ± 5
							AMP (W·kg-1)	19 ± 2		14 ± 1		13 ± 1					12 ± 1

						Midfielder	Total distance (m/min)	196 ± 22		145 ± 15		145 ± 15					135 ± 15
							HMLD (m/min)	68 ± 15		38 ± 8		32 ± 7					26 ± 6
							AMP (W·kg-1)	19 ± 2		15 ± 1		14 ± 1					13 ± 1

						Off. Midfielder	Total distance (m/min)	194 ± 25		143 ± 12		143 ± 12					132 ± 12
							HMLD (m/min)	74 ± 14		44 ± 8		32 ± 7					30 ± 6
							AMP (W·kg-1)	19 ± 2		15 ± 1		14 ± 1					13 ± 1

						Forward	Total distance (m/min)	184 ± 25		144 ± 15		133 ± 13					122 ± 13
							HMLD (m/min)	66 ± 16		38 ± 9		31 ± 7					25 ± 6
							AMP (W·kg-1)	17 ± 2		13 ± 2		12 ± 1					11 ± 1

						Whole team (Average)	Total distance (m/min)	179 ± 24		139 ± 17		139 ± 13					118 ± 14
							HMLD (m/min)	69 ± 16		39 ± 8		32 ± 7					26 ± 5
							AMP (W·kg-1)	18 ± 2		14 ± 1		13 ± 1					12 ± 1

Fereday et al. [12]	GPS (10 Hz; Optim-eye S5, Catapult Sports, Melbourne, Australia)	PRO (English) 25 ± 4	25	28	W	Defenders	Total distance (m/min)	188 ± 19	155 ± 14	143 ± 11	136 ± 11	131 ± 11	128 ± 10	125 ± 10	122 ± 10	120 ± 9	119 ± 9
							HSR (m/min)	60 ± 21	34 ± 16	27 ± 13	23 ± 12	20 ± 10	18 ± 9	16 ± 8	15 ± 7	14 ± 7	13 ± 6

						Midfielders	Total distance (m/min)	196 ± 19	163 ± 16	150 ± 15	143 ± 14	138 ± 14	134 ± 14	131 ± 13	129 ± 13	127 ± 13	125 ± 13
							HSR (m/min)	61 ± 6	38 ± 20	30 ± 16	25 ± 14	22 ± 13	20 ± 11	18 ± 10	17 ± 9	16 ± 8	15 ± 7

						Forwards	Total distance (m/min)	180 ± 19	149 ± 15	139 ± 15	131 ± 15	127 ± 14	124 ± 15	122 ± 15	119 ± 14	117 ± 14	116 ± 14
							HSR (m/min)	56 ± 19	34 ± 12	27 ± 11	22 ± 10	20 ± 8	18 ± 7	16 ± 6	15 ± 6	14 ± 5	14 ± 5

						Whole team (Average)	Total distance (m/min)	190 ± 20	157 ± 17	145 ± 15	138 ± 14	133 ± 14	130 ± 14	127 ± 13	125 ± 13	123 ± 7	121 ± 13
							HSR (m/min)	60 ± 23	36 ± 17	28 ± 14	24 ± 12	21 ± 11	19 ± 10	17 ± 9	16 ± 8	15 ± 7	14 ± 6

Martín-García et al. [23]	GPS (Viper Pod, 50 g, 88 x 33 mm, STATSports Viper, Northern Ireland)	PRO (Spanish) 20 ± 2	23	37	W	Wide defender	Total distance (m/min)	195 ± 16		152 ± 9		139 ± 8					128 ± 8
							HSR (m/min)	47 ± 24		20 ± 9		15 ± 6					11 ± 4
							Sprint (m/min)	14 ± 17		4 ± 5		3 ± 3					3 ± 2
							AMP (W·kg-1)	19 ± 2		14 ± 1		13 ± 1					12 ± 1
							HMLD (m/min)	70 ± 18		40 ± 9		33 ± 6					28 ± 5
							Acc (n)	3 ± 1		2 ± 1		2 ± 1					2 ± 1
							Dec (n)	4 ± 1		3 ± 1		3 ± 1					2 ± 1
							HI Acc/Dec	7 ± 3		5 ± 2		5 ± 1					5 ± 1

						Central Defender	Total distance (m/min)	182 ± 16		143 ± 10		133 ± 8					122 ± 7
							HSR (m/min)	35 ± 24		11 ± 9		8 ± 5					6 ± 3
							Sprint (m/min)	12 ± 19		3 ± 5		1 ± 2					1 ± 1
							AMP (W·kg-1)	17 ± 2		13 ± 1		12 ± 1					11 ± 1
							HMLD (m/min)	59 ± 17		31 ± 8		25 ± 5					22 ± 4
							Acc (n)	3 ± 1		2 ± 1		2 ± 1					2 ± 0
							Dec (n)	3 ± 2		3 ± 1		2 ± 1					2 ± 1
							HI Acc/Dec	6 ± 3		5 ± 2		4 ± 1					4 ± 1

						Midfielder	Total distance (m/min)	204 ± 15		161 ± 8		140 ± 8					140 ± 7
							HSR (m/min)	30 ± 22		12 ± 7		7 ± 3					7 ± 3
							Sprint (m/min)	6 ± 11		2 ± 3		1 ± 1					1 ± 1
							AMP (W·kg-1)	19 ± 1		15 ± 1		14 ± 1					13 ± 1
							HMLD (m/min)	66 ± 16		38 ± 7		32 ± 5					27 ± 5
							Acc (n)	3 ± 1		3 ± 1		3 ± 1					3 ± 1
							Dec (n)	3 ± 1		3 ± 1		3 ± 1					3 ± 1
							HI Acc/Dec	6 ± 2		6 ± 2		6 ± 1					5 ± 1

						Wide midfielder	Total distance (m/min)	201 ± 20		157 ± 16		146 ± 16					135 ± 16
							HSR (m/min)	36 ± 20		15 ± 8		11 ± 5					9 ± 4
							Sprint (m/min)	7 ± 12		2 ± 3		2 ± 2					1 ± 2
							AMP (W·kg-1)	19 ± 2		15 ± 1		14 ± 1					13 ± 1
							HMLD (m/min)	70 ± 16		39 ± 10		34 ± 9					29 ± 8
							Acc (n)	3 ± 2		3 ± 1		3 ± 1					2 ± 1
							Dec (n)	4 ± 2		3 ± 1		3 ± 1					3 ± 1
							HI Acc/Dec	7 ± 3		6 ± 2		5 ± 2					5 ± 1

						Forward	Total distance (m/min)	181 ± 20		138 ± 16		128 ± 14					127 ± 13
							HSR (m/min)	38 ± 22		17 ± 9		13 ± 6					11 ± 4
							Sprint (m/min)	11 ± 14		4 ± 4		3 ± 3					2 ± 2
							AMP (W·kg-1)	18 ± 2		13 ± 2		12 ± 1					11 ± 1
							HMLD (m/min)	62 ± 18		36 ± 10		29 ± 8					25 ± 6
							Acc (n)	3 ± 2		2 ± 1		2 ± 1					2 ± 1
							Dec (n)	3 ± 2		3 ± 1		2 ± 1					2 ± 1
							HI Acc/Dec	6 ± 3		5 ± 2		4 ± 2					4 ± 2

						Whole team (Average)	Total distance (m/min)	192 ± 20		149 ± 15		138 ± 14					127 ± 13
							HSR (m/min)	38 ± 23		16 ± 9		12 ± 6					9 ± 4
							Sprint (m/min)	11 ± 16		3 ± 4		2 ± 3					2 ± 2
							AMP (W·kg-1)	18 ± 2		14 ± 1		13 ± 1					12 ± 1
							HMLD (m/min)	65 ± 18		37 ± 9		31 ± 7					26 ± 6
							Acc (n)	3 ± 2		2 ± 1		2 ± 1					2 ± 1
							Dec (n)	3 ± 2		3 ± 1		3 ± 1					2 ± 1
							HI Acc/Dec	6 ± 3		5 ± 2		5 ± 2					5 ± 1

Martín-García et al. [24]	GPS units (Viper Pod, 50 g, 88 x 33 mm, STATSports Viper, Northern Ireland)	PRO (Spanish reserve squad) 20 ± 1	**21**	**37**	**W**	Central defender	Total Distance (m/min)										101 ± 6
							HSR (m/min)										5 ± 1
							Sprint (m/min)										1 ± 1
							HMLD(m/min)										16 ± 2
							AMP (W·kg-1)										9 ± 1
							ACC (n/min)										2 ± 0.2
							DEC (n/min)										2 ± 0.2

						Wide defender	Total Distance (m/min)										105 ± 6
							HSR (m/min)										7 ± 2
							Sprint (m/min)										2 ± 1
							HMLD(m/min)										19 ± 3
							AMP (W·kg-1)										10 ± 1
							ACC (n/min)										2 ± 0.4
							DEC (n/min)										2 ± 0.3

						Midfielder	Total Distance (m/min)										115 ± 8
							HSR (m/min)										5 ± 2
							Sprint (m/min)										1 ± 1
							HMLD(m/min)										19 ± 3
							AMP (W·kg-1)										11 ± 1
							ACC (n/min)										2 ± 0.4
							DEC (n/min)										2 ± 0.4

						Offensive midfielder	Total Distance (m/min)										114 ± 9
							HSR (m/min)										6 ± 2
							Sprint (m/min)										1 ± 1
							HMLD(m/min)										23 ± 5
							AMP (W·kg-1)										11 ± 1
							ACC (n/min)										2 ± 0.4
							DEC (n/min)										2 ± 0.4

						Forward	Total Distance (m/min)										96 ± 11
							HSR (m/min)										7 ± 3
							Sprint (m/min)										2 ± 1
							HMLD(m/min)										16 ± 4
							AMP (W·kg-1)										9 ± 1
							ACC (n/min)										1 ± 1
							DEC (n/min)										2 ± 1

						Whole team (Average)	Total Distance (m/min)										105 ± 11
							HSR (m/min)										6 ± 2
							Sprint (m/min)										1 ± 1
							HMLD(m/min)										18 ± 4
							AMP (W·kg-1)										10 ± 1
							ACC (n/min)										2 ± 1
							DEC (n/min)										2 ± 0.4

Oliva-Lozano et al. [13]	GPS (WIMU Pro, RealTrack Systems, Almeria, Spain)	PRO (Spanish 2nd league) 26.8 ± 3.8	20	13	W	Central Defender	Total distance (m/min)	187 ± 22		142 ± 7		130 ± 7					119 ± 7
							HSR (m/min)	52 ± 9		25 ± 5		18 ± 4					12 ± 2
							Sprint (m/min)	27 ± 7		10 ± 3		7 ± 2					4 ± 2
							Acc (n/min)	3 ± 0.6		2 ± 0.2		1 ± 0.2					1 ± 0.2
							Dec (n/min)	4 ± 0.5		2 ± 0.3		2 ± 0.2					1 ± 0.2

						Wide Defender	Total distance (m/min)	207 ± 14		159 ± 8		143 ± 8					131 ± 7
							HSR (m/min)	69 ± 10		34 ± 5		25 ± 4					18 ± 3
							Sprint (m/min)	34 ± 10		14 ± 4		10 ± 3					6 ± 2
							Acc (n/min)	4 ± 0.9		2 ± 0.4		2 ± 0.3					1 ± 0.2
							Dec (n/min)	5 ± 0.7		3 ± 0.3		2 ± 0.3					2 ± 0.2

						Wide Midfielder	Total distance (m/min)	203 ± 13		156 ± 9		145 ± 13					162 ± 37
							HSR (m/min)	71 ± 16		34 ± 7		27 ± 6					19 ± 6
							Sprint (m/min)	39 ± 14		17 ± 6		11 ± 4					8 ± 3
							Acc (n/min)	4 ± 0.7		2 ± 0.3		2 ± 0.3					1 ± 0.4
							Dec (n/min)	5 ± 0.7		3 ± 0.5		2 ± 0.4					2 ± 0.5

						Midfielder	Total distance (m/min)	202 ± 17		158 ± 8		147 ± 9					132 ± 40
							HSR (m/min)	53 ± 12		25 ± 6		19 ± 5					13 ± 4
							Sprint (m/min)	21 ± 8		7 ± 3		5 ± 2					3 ± 1
							Acc (n/min)	4 ± 0.6		2 ± 0.4		2 ± 0.3					1 ± 0.4
							Dec (n/min)	4 ± 0.9		2 ± 0.5		2 ± 0.5					2 ± 0.5

						Forward	Total distance (m/min)	206 ± 18		156 ± 15		144 ± 46					132 ± 52
							HSR (m/min)	62 ± 13		29 ± 7		22 ± 7					16 ± 6
							Sprint (m/min)	30 ± 9		12 ± 4		8 ± 3					4 ± 2
							Acc (n/min)	3 ± 0.7		2 ± 0.5		1 ± 0.5					1 ± 0.4
							Dec (n/min)	4 ± 0.9		2 ± 0.6		2 ± 0.6					1 ± 0.6

						Whole team (Average)	Total distance (m/min)	201 ± 18		155 ± 11		142 ± 25					130 ± 37
							HSR (m/min)	61 ± 15		30 ± 8		22 ± 6					16 ± 6
							Sprint (m/min)	30 ± 12		12 ± 5		8 ± 4					5 ± 2
							Acc (n/min)	4 ± 0.7		2 ± 0.4		2 ± 0.3					1 ± 0.3
							Dec (n/min)	5 ± 0.8		2 ± 0.5		2 ± 0.5					2 ± 0.5

Oliva-Lozano et al. [26]	GPS (WIMU Pro, RealTrack Systems, Almeria, Spain)	PRO (Spanish 2nd league) 26.78 ± 3.77	23	13	W	Whole team (Average) Short micro	Total distance (m/min)	198 ± 23		156 ± 20		145 ± 17					133 ± 16
							HSR (m/min)	61 ± 1		29 ± 8		25 ± 7					16 ± 5
							Sprint (m/min)	32 ± 13		12 ± 6		8 ± 4					5 ± 3

						Whole team (Average) Regular micro	Total distance (m/min)	217 ± 65		157 ± 26		141 ± 16					130 ± 12
							HSR (m/min)	61 ± 19		28 ± 8		23 ± 6					15 ± 5
							Sprint (m/min)	28 ± 14		12 ± 7		8 ± 5					5 ± 3

						Whole team (Average) Long micro	Total distance (m/min)	201 ± 15		157 ± 13		146 ± 12					132 ± 9
							HSR (m/min)	63 ± 14		32 ± 8		25 ± 6					17 ± 4
							Sprint (m/min)	33 ± 12		13 ± 5		9 ± 4					5 ± 2

Oliva-Lozano et al. [10]	GPS (WIMU Pro, RealTrack Systems, Almeria, Spain)	PRO (Spanish 2nd league) 26.8 ± 3.8	23	13	W	Central defender	Total distance (m/min)	169 ± 32		133 ± 21		122 ± 17					109 ± 15
							HSR (m/min)	53 ± 34		18 ± 11		13 ± 8					9 ± 6
							Sprint (m/min)	17 ± 11		6 ± 5		4 ± 3					3 ± 2

					W	Forward	Total distance (m/min)	186 ± 61		146 ± 26		133 ± 20					119 ± 17
							HSR (m/min)	60 ± 128		21 ± 10		16 ± 8					11 ± 6
							Sprint (m/min)	19 ± 14		7 ± 5		5 ± 4					3 ± 2

					W	Wide defender	Total distance (m/min)	188 ± 32		149 ± 22		135 ± 19					119 ± 15
							HSR (m/min)	67 ± 137		25 ± 11		19 ± 9					13 ± 6
							Sprint (m/min)	22 ± 13		9 ± 7		6 ± 5					4 ± 3

					W	Midfielder	Total distance (m/min)	202 ± 142		154 ± 60		141 ± 39					125 ± 25
							HSR (m/min)	65 ± 159		22 ± 43		16 ± 26					11 ± 14
							Sprint (m/min)	29 ± 14		10 ± 5		7 ± 3					4 ± 1

					W	Wide midfielder	Total distance (m/min)	188 ± 42		145 ± 21		132 ± 119					118 ± 16
							HSR (m/min)	61 ± 96		26 ± 10		20 ± 8					14 ± 6
							Sprint (m/min)	26 ± 16		11 ± 7		8 ± 5					5 ± 3

					1st	Whole team	Total distance (m/min)	217 ± 158		160 ± 58		145 ± 36					132 ± 20

					2nd	Whole team	Total distance (m/min)	191 ± 24		147 ± 16		136 ± 14					124 ± 12

Riboli et al. [27]	Semi-automatic tracking system (Stats Perform, Chicago, Illinois, USA)	PRO (Italian)	148	46	W												

Riboli et al. [15]	Semi-automatic tracking system (Stats Perform, Chicago, Illinois, USA)	PRO (Italian)	**223**	**18**	**W**	Central defender	Total distance (m/min)	181 ± 30	151 ± 28	141 ± 23	136 ± 26	133 ± 23					121 ± 28
							HSR (m/min)	50 ± 22	23 ± 11	18 ± 14	18 ± 12	17 ± 7					12 ± 3
							Very HSR (m/min)	34 ± 11	19 ± 7	15 ± 5	13 ± 4	11 ± 4					8 ± 3
							Sprint (m/min)	36 ± 15	19 ± 9	14 ± 6	11 ± 5	10 ± 4					6 ± 3
							Acc/Dec (n/min)	31 ± 4	18 ± 3	15 ± 2	12 ± 2	11 ± 2					7 ± 1
							AMP (W.kg-1)	19 ± 4	16 ± 3	14 ± 2	14 ± 2	13 ± 3					12 ± 2
							HMLD(m/min)	88 ± 20	60 ± 13	52 ± 11	46 ± 10	42 ± 12					36 ± 9

						Wide defender	Total distance (m/min)	187 ± 27	157 ± 27	144 ± 21	140 ± 23	136 ± 21					121 ± 30
							HSR (m/min)	56 ± 19	26 ± 14	24 ± 19	21 ± 14	19 ± 6					13 ± 3
							Very HSR (m/min)	37 ± 13	22 ± 8	17 ± 6	14 ± 5	12 ± 4					9 ± 4
							Sprint (m/min)	44 ± 15	23 ± 9	18 ± 7	14 ± 6	11 ± 5					7 ± 3
							Acc/Dec (n/min)	33 ± 5	20 ± 3	15 ± 2	13 ± 2	11 ± 2					8 ± 1
							AMP (W.kg-1)	20 ± 3	16 ± 3	14 ± 2	14 ± 3	12 ± 4					12 ± 3
							HMLD(m/min)	92 ± 24	65 ± 17	54 ± 13	49 ± 13	43 ± 16					38 ± 13

						Midfielder	Total distance (m/min)	198 ± 27	168 ± 28	156 ± 24	150 ± 26	145 ± 24					130 ± 33
							HSR (m/min)	68 ± 19	36 ± 12	36 ± 16	32 ± 12	27 ± 5					21 ± 4
							Very HSR (m/min)	39 ± 12	24 ± 8	19 ± 5	16 ± 5	14 ± 4					10 ± 3
							Sprint (m/min)	40 ± 17	23 ± 10	16 ± 7	13 ± 6	11 ± 5					7 ± 3
							Acc/Dec (n/min)	31 ± 4	18 ± 2	15 ± 2	12 ± 2	11 ± 2					8 ± 1
							AMP (W.kg-1)	21 ± 4	17 ± 2	16 ± 2	15 ± 2	13 ± 5					13 ± 3
							HMLD(m/min)	103 ± 17	75 ± 14	64 ± 10	59 ± 11	50 ± 18					46 ± 11

						Wide midfielder	Total distance (m/min)	198 ± 19	167 ± 18	157 ± 14	148 ± 23	143 ± 19					126 ± 37
							HSR (m/min)	68 ± 20	35 ± 13	34 ± 17	32 ± 14	26 ± 6					23 ± 5
							Very HSR (m/min)	41 ± 14	25 ± 9	20 ± 6	17 ± 6	15 ± 5					11 ± 5
							Sprint (m/min)	49 ± 17	27 ± 10	20 ± 8	16 ± 6	15 ± 6					9 ± 4
							Acc/Dec (n/min)	35 ± 4	21 ± 3	17 ± 2	14 ± 2	13 ± 1					9 ± 2
							AMP (W.kg-1)	22 ± 8	17 ± 5	16 ± 3	15 ± 3	13 ± 4					13 ± 3
							HMLD(m/min)	103 ± 21	75 ± 16	64 ± 13	57 ± 13	50 ± 18					45 ± 12

						Forward	Total distance (m/min)	177 ± 38	148 ± 34	139 ± 30	132 ± 31	129 ± 30					108 ± 43
							HSR (m/min)	48 ± 21	23 ± 13	18 ± 17	20 ± 13	13 ± 6					23 ± 5
							Very HSR (m/min)	34 ± 13	22 ± 8	16 ± 6	14 ± 5	12 ± 5					9 ± 4
							Sprint (m/min)	38 ± 19	21 ± 11	16 ± 8	13 ± 8	11 ± 6					7 ± 4
							Acc/Dec (n/min)	29 ± 5	17 ± 3	14 ± 2	12 ± 2	11 ± 2					7 ± 2
							AMP (W.kg-1)	19 ± 4	16 ± 3	14 ± 3	13 ± 3	11 ± 6					11 ± 3
							HMLD(m/min)	86 ± 23	60 ± 17	52 ± 13	46 ± 13	36 ± 20					35 ± 13

						Wide forward	Total distance (m/min)	191 ± 19	160 ± 13	150 ± 12	143 ± 12	138 ± 10					126 ± 15
							HSR (m/min)	58 ± 19	29 ± 10	26 ± 14	24 ± 11	21 ± 5					15 ± 3
							Very HSR (m/min)	39 ± 8	22 ± 6	18 ± 4	14 ± 4	13 ± 3					10 ± 3
							Sprint (m/min)	46 ± 14	27 ± 8	19 ± 7	16 ± 6	13 ± 5					8 ± 3
							Acc/Dec (n/min)	33 ± 4	21 ± 2	16 ± 2	14 ± 1	12 ± 1					8 ± 1
							AMP (W·kg-1)	20 ± 2	16 ± 2	15 ± 1	14 ± 1	13 ± 3					12 ± 2
							HMLD(m/min)	94 ± 17	66 ± 12	56 ± 9	51 ± 9	44 ± 13					39 ± 8

						Whole team (Average)	Total distance (m/min)	188 ± 25	159 ± 24	148 ± 20	141 ± 22	137 ± 43					122 ± 28
							HSR (m/min)	58 ± 17	128 ± 12	26 ± 16	25 ± 12	21 ± 6					16 ± 3
							Very HSR (m/min)	37 ± 11	22 ± 7	17 ± 5	14 ± 5	13 ± 5					9 ± 3
							Sprint (m/min)	42 ± 16	23 ± 9	17 ± 7	14 ± 6	12 ± 6					7 ± 3
							Acc/Dec (n/min)	32 ± 7	19 ± 4	15 ± 3	13 ± 2	11 ± 3					8 ± 2
							AMP (W·kg-1)	20 ± 4	16 ± 3	15 ± 2	14 ± 2	12 ± 4					12 ± 3
							HMLD(m/min)	94 ± 20	67 ± 15	57 ± 12	51 ± 11	44 ± 16					40 ± 11

Note: Off: offensive; AMP: average metabolic power; HI: high intensity; HML: high metabolic load distance (> 20 W.kg^-1^); HMLD: high metabolic load distance; HSR: high-speed running; HMP: high metabolic power; Acc: acceleration; Dec: deceleration; M = n of matches; P = n of players; W = whole match; ND: non-described.

**TABLE 4:** Performance indicators during most demanding passages in professional women players.

Ref.	EPTS	Level (League) Age	N		Half	Playing position	Variable	Epochs (values per minutes)		
			P	M				1	5	10
Muñiz- González et al. [25]	GPS (CatapultSports®, GPSports EVO®, Canberra, Australia),	Female PRO (Spanish) 24.2 ± 6.3	18	15	W	Central defender	Distance (m/min)	153 ± 11	116 ± 6	107 ± 9
							HSR (m/min)	26 ± 6	6 ± 2	3 ± 1
							HMLD	61 ± 8	28 ± 3	23 ± 3
							Acc (m/s)	0.86 ± 0	0.66 ± 0	0.61 ± 0

						Wide defender	Distance(m/min)	161 ± 13	119 ± 8	109 ± 10
							HSR (m/min)	30 ± 9	9 ± 2	5 ± 1
							HMLD	71 ± 13	33 ± 6	27 ± 6
							Acc (m/s)	0.95 ± 0	0.74 ± 0	0.68 ± 0

						Midfielder	Distance(m/min)	163 ± 11	125 ± 11	116 ± 11
							HSR (m/min)	21 ± 8	4 ± 1	2 ± 0
							HMLD	66 ± 10	33 ± 6	28 ± 4
							Acc (m/s)	0.87 ± 0	0.67 ± 0	0.62 ± 0

						Wide midfielder	Distance(m/min)	158 ± 11	118 ± 11	109 ± 12
							HSR (m/min)	34 ± 8	9 ± 2	6 ± 1
							HMLD	67 ± 8	33 ± 5	27 ± 4
							Acc (m/s)	0.95 ± 0	0.75 ± 0	0.67 ± 0

						Forward	Distance(m/min)	152 ± 15	117 ± 12	107 ± 11
							HSR (m/min)	32 ± 7	9 ± 2	5 ± 1
							HMLD	67 ± 9	33 ± 5	27 ± 4
							Acc (m/s)	0.88 ± 0	0.67 ± 0	0.61 ± 0

						Whole team (Average)	Distance(m/min)	157 ± 12	119 ± 10	110 ± 11
							HSR (m/min)	29 ± 8	7 ± 2	4 ± 1
							HMLD	66 ± 10	32 ± 5	26 ± 4
							Acc (m/s)	0.9 ± 0	0.7 ± 0	0.6 ± 0

Trewin et al. [28]	GPS (Minimax S4, Catapult Inno-vations, Australia)	PRO (ND)	45	55	W	Wide defender	TD (m/min)		144 ± 9	
							LSR (m/min)			
							HSR (m/min)		31 ± 8	
							Acc (count/min)		4 ± 0.5	
							HSR (count/min)			
							Sprint (count/min)			
							PL (AU/min)		14 ± 2	

						Central defender	TD (m/min)		132 ± 10	
							LSR (m/min)			
							HSR (m/min)		20 ± 9	
							Acc (count/min)		3 ± 0.6	
							HSR (count/min)			
							Sprint (count/min)			
							PL (AU/min)		14 ± 2	

						Midfield	TD (m/min)		146 ± 10	
							LSR (m/min)			
							HSR (m/min)		25 ± 7	
							Acc (count/min)		3 ± 0.5	
							HSR (count/min)			
							Sprint (count/min)			
							PL (AU/min)		18 ± 3	

						Forward	TD (m/min)		141 ± 12	
							LSR (m/min)			
							HSR (m/min)		25 ± 6	
							Acc (count/min)		3 ± 0.7	
							HSR (count/min)			
							Sprint (count/min)			
							PL (AU/min)		14 ± 3	

						Whole team (Average)	TD (m/min)		141 ± 12	
							LSR (m/min)			
							HSR (m/min)		25 ± 8	
							Acc (count/min)		3 ± 0.6	
							HSR (count/min)			
							Sprint (count/min)			
							PL (AU/min)		15 ± 3	

Note: AMP: average metabolic power; HI: high intensity; HMLD: high metabolic load distance; HSR: high-speed running; HMP: high metabolic power; Acc: acceleration; PL: player load; M = n of matches; P = n of players; W = whole match.

**FIG. 2:**
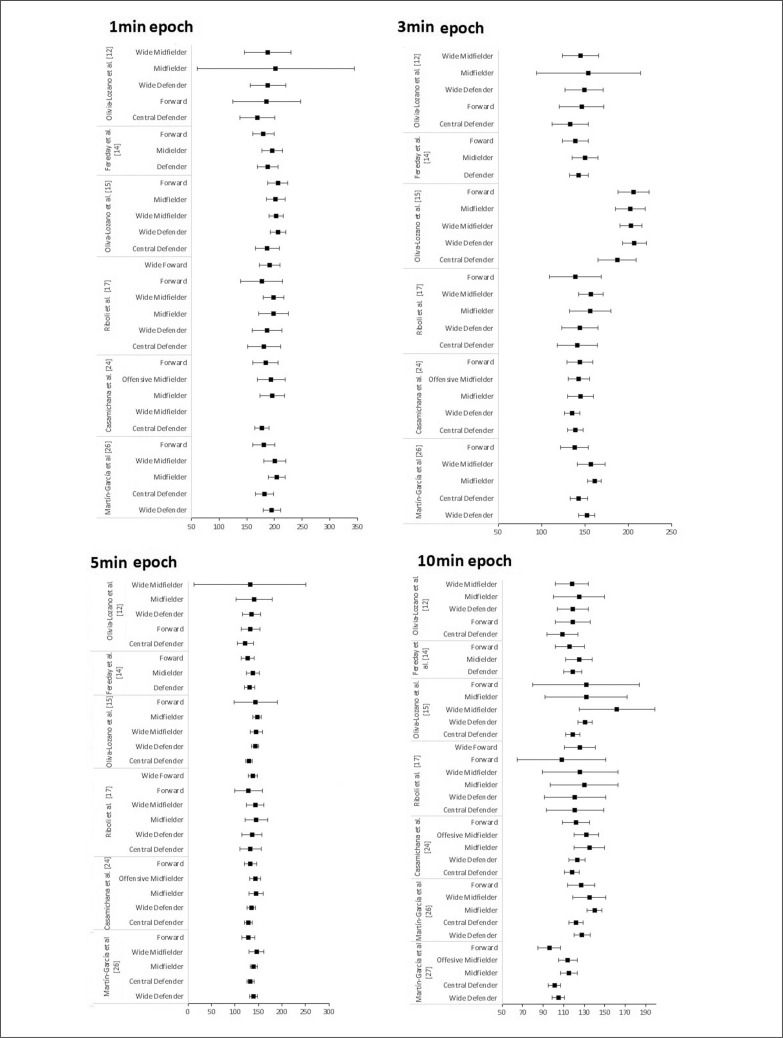
Peak demands of relative total distance (m/min) during 1-, 3-, 5- and 10-min time windows among professional men player positions.

**FIG. 3:**
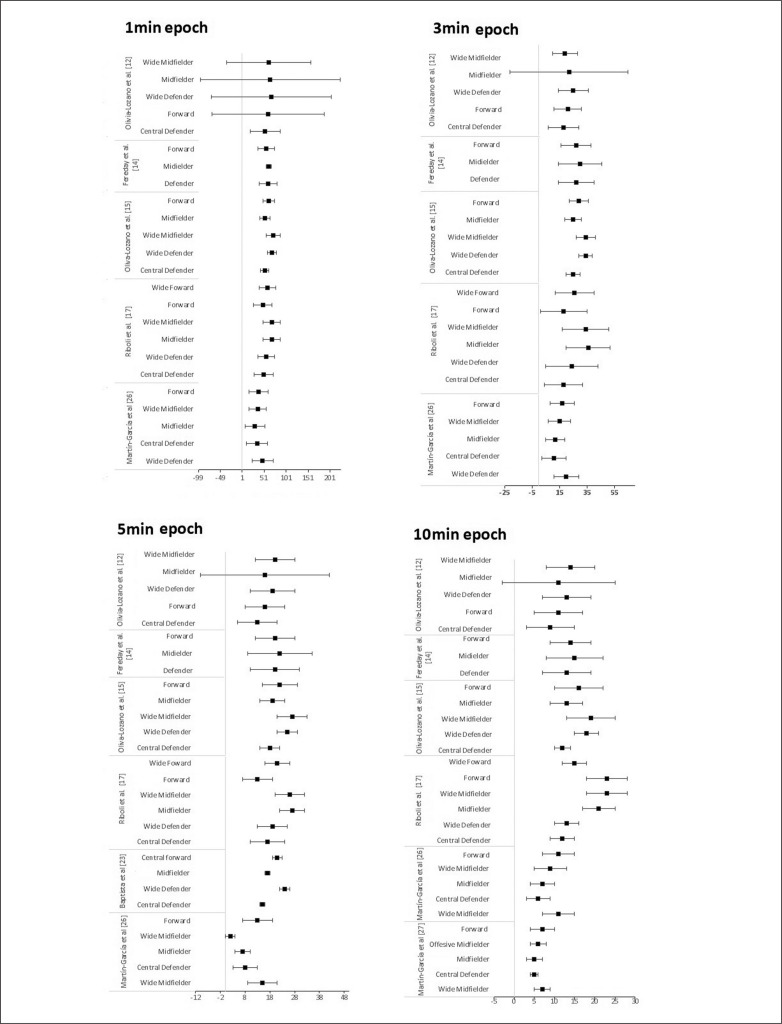
Peak demands of high-speed running distance (HRS, m/min) during 1-, 3-, 5- and 10-min time windows among professional men player positions.

**FIG. 4:**
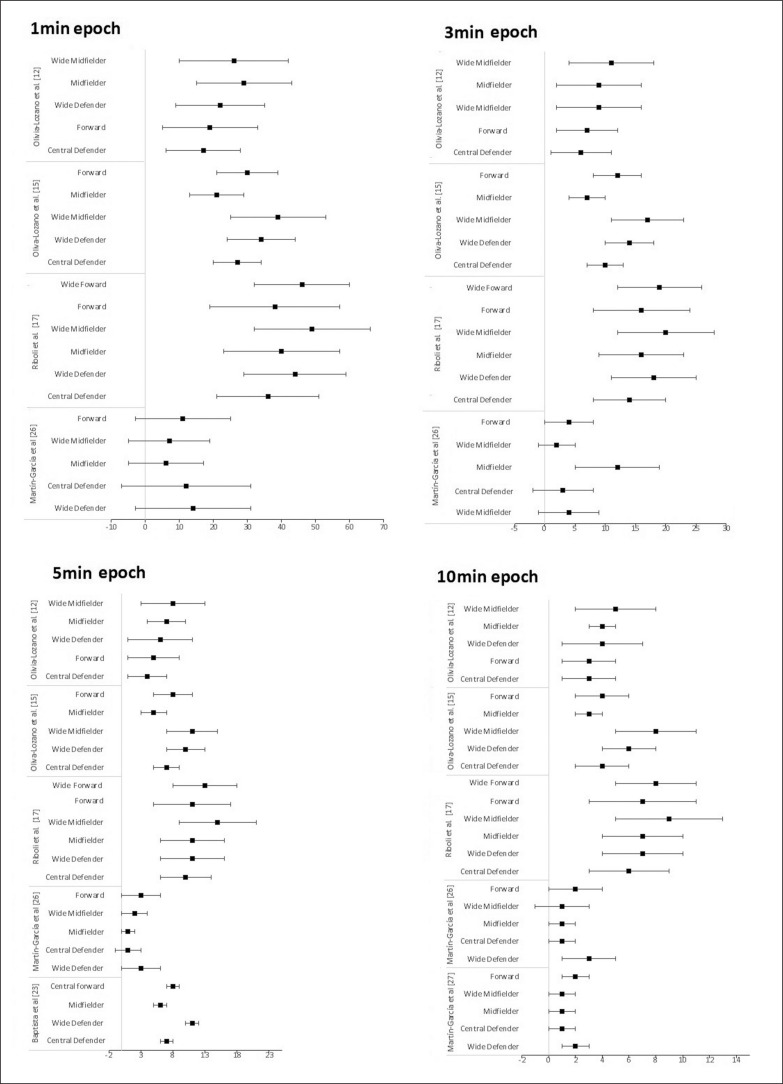
Peak demands of sprint (m/min) during 1-, 3-, 5- and 10-min time windows among professional men player positions.

Figures 2–4 showed data for the most variables (total distance, high-speed running, sprint) and epochs (1, 3, 5 and 10 min) used in the studies analysed. Fig 2 presents the WCS of relative total distance during 1-, 3-, 5- and 10-min epoch between professional male player positions for the studies [10, 12, 13, 15, 23].

Figure 3 presents the WCS of high-speed running during 1-, 3-, 5- and 10-min epoch between professional male player positions for the studies for the studies [10, 12, 13, 15, 23].

Figure 4 presents the WCS of sprint during 1-, 3-, 5- and 10-min epoch between professional male player positions for the studies for the studies [10, 13, 15, 23].

**FIG. 5:**
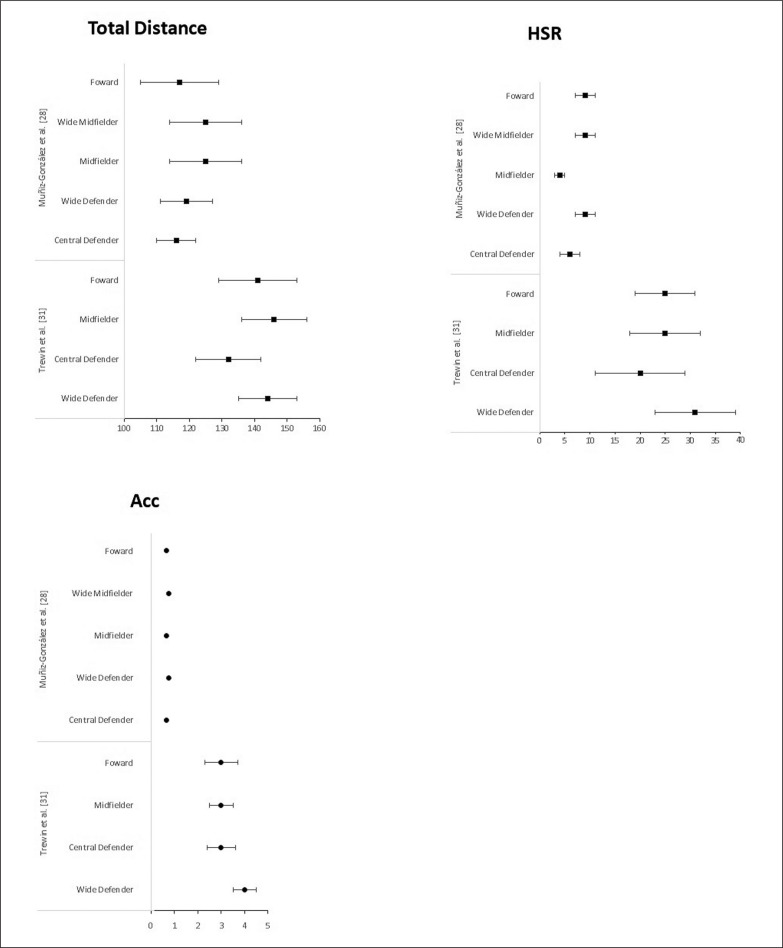
Peak demands of relative total distance (m/min) high-speed running distance (HSR, m/min) and acceleration (Acc, n/min) during 5 min time windows among professional women player positions.

Figure 5 presents the peak demands of relative total distance, high-speed running and acceleration during 5 min epoch between professional women player positions for both studies [25, 28].

## DISCUSSION

The main purpose of this systematic review was to summarize the evidence about WCS in professional soccer. The literature searches and screening steps were focused mainly on papers that have made comparisons including independent measures such as epochs (time windows), playing positions, sexes, and contextual factors within the aforementioned umbrella research topic. A total of 12 articles were reviewed and, in some cases, their pooled results pointed in the same direction. The main findings were: (i) there was an inverse relationship between the duration of WCS windows and running output during match play; (ii) the main differences were found between 1-min epochs and the remaining time windows; (iii) occurrences of WCS during soccer matches were also position-dependent across studies, especially, analyzing total distance covered; (iv) although these conclusions remain in articles that considered women soccer players, different values were reported between sexes; (v) contextual constraints (match status, team formation, and match location) should be considered to understand WCS in soccer.

In soccer, two teams face one another with exactly opposite aims, leading players to organize themselves with teammates and consider the opponents´ behavior. In this scenario, a player´s movement (teammate or opponent) induces another player´s adaptation, which subsequently provokes the adaptation of another. In this respect, soccer has been highlighted as a game involving continuous adaptations and re-adaptations [29]. Considering this concept, the team with possession shares the ball through passes between teammates, who move along the field providing solutions for the player with the ball [30]. This collective behavior of the attacking team is counteracted by individual defending player behavior, combining into collective defending behavior, confronting each other opposite strategies. In this scenario, while team-to-team, or specifically, player-to-player synchronization remains, a steady state dominates [31]. However, when one of the attacking player’s movements (usually high-intensity movement) breaks player-to-player synchronization, the remaining players leave their position to alleviate the defensive imbalance, trying to recover the general equilibrium [31]. Therefore, if the defending team can stop the progression of the attacking team towards its goal, the steady state will be regained, while if the defending team cannot, a chaotic situation will arise [31], leading to WCS. In soccer literature, it has been highlighted that the relationship between players´ physical fitness and ranking outcomes is poor [32, 33], emphasizing teams´ technical and tactical effectiveness rather than high levels of physical performance [33]. However, further studies could consider if the player´s physical condition may be more relevant in WCS. To date, the WCS have been analyzed using some factors that have influenced the extracted outcomes. Therefore, to analyze the influence of each of them may be of interest for soccer community.

### Worst-case scenarios depending on the time window

Since high-intensity actions have been included in the principal components that explained players´ performance [34, 35], interest in analyzing WCS has grown exponentially. Since different authors have found the influence of different epochs in running outputs (Table 3 and 4), the definition of WCS has led researchers to redefine this concept understanding the WCS as situations that demand maximal physical load in a given time window [36]. Thus, the analysis of WCS in different time windows has been the first aim of this systematic review.

In soccer literature on WCS, time windows between 1 min and 10 min have been considered, highlighting differences among them. For example, men soccer players travel from 132 to 233 m/min, from 17 to 55 m/min at high-speed running distance (HSR), from 7 to 29 m/min at sprint intensity, from 2 to 5 acc and dec/min, from 30 to 72 m/min of HMLD, and values from 11 to 19 W·kg^-1^ of AMP. When considering these values as a reference for training task design, there may be a lack of precision, making it difficult to interpret standard values for accurate design. In this scenario, physical fitness and conditioning coaches, together with head coaches, should consider the team´s characteristics and the game styles, which could define greater or shorter most demanding passages. To date, game styles may be divided into (i) direct attack and deep defending, (ii) direct attack and high-pressure defending, (iii) elaborate attack and high-pressure defending, and, (iv) elaborate attack and deep defending [37]. In this regard, if the attacking team breaks the defending team players´ synchronization while they maintain high-pressure defending, longer WCS will be expected from the breakdown of the steady state on one side of the field, to the goal scoring situation on the other side. On the contrary, due to the distance from the area where the order is broken to the goal-scoring zone, deep defending may induce shorter WCS. However, these hypotheses that may encourage the individualization of time windows depending on team characteristics should be considered with caution due to the chaotic nature of soccer games.

The most commonly considered epochs were 1-, 3-, 5-, and 10-min time windows. Among them, large differences appear between the analysis of WCS using 1 min time windows in comparison with the following one, in this case 2-min. For example, the analysis reported that wide defenders travel different distances for a time window of 1 min (233 m/min), 3 min (157 m/min), 5 min (138 m/min), and 10 min (121 m/min). In addition, these players at HSR showed differences greater than 50% between the 1 min (55 m/min) and 3 min (26 m/min), while for 5 min (20 m/min) and 10 min (12 m/min) time windows, differences were slight. But, in addition to the present example, these differences between 1 min and 3 min time-windows remain wide, independently of playing position, for variables such as sprint (29 m/min and 11 m/min), Acc (4 n/min and 2 n/min), and HMLD (72 m/min and 41 m/min), respectively; while the differences between the remaining time-windows (3, 5, and 10 min) were lower than 10 m/min, with the exception of total distance covered that showed differences of up to 30 m/min between 3 min and 5 min time-windows. Although these large differences appear between 1 min and 2 min time windows, the limited number of studies that considered 2 min epochs mean that this conclusion requires more research.

In brief, from approximately 90% of the studies that compare values from WCS in different time windows, a clear trend reveals that the smaller the time window, the higher the values reported, especially between 1 min and the remaining time widows.

### Worst-case scenarios depending on playing position

Since the literature reveals that soccer players´ performance varies depending on their positional roles (central defender (CD), wide defender (WD), midfielder (MD), wide midfielder (WM), and forward (F)) [38, 39], it was expected that players´ performance may differ during WCS.

In soccer literature, it has been highlighted that MD traveled the longest average distance during competition, followed by F and defenders [33, 40, 41]. Regarding HSR, full-backs, MD, and advanced MD covered a greater distance, while CD and F covered more distance at high power [42]. In addition, the full-backs and advanced MD produce more accelerations and decelerations in comparison with the other roles, while MD usually develop greater metabolic power [42]. Accordingly, WM (from 135 to 233 m/min), MD (from 126 to 200 m/min), and WM (from 121 to 233 m/min) are the players that traveled a longer total distance during WCS, followed by F (from 117 to 186 m/min) and CD (from 115 to 179 m/min). However, there were not wide differences among playing positions in other variables that describe high-intensity efforts. For example, CD and F, who perform a lower distance at high intensity [42], perform between 9 and 51 m/min in HSR, while WD, WM, and MD, who record greater values of HSR in a whole match [42], perform between 12 and 57 m/min. Similarly, WM and MD perform greater distances at sprint intensity (from 4 to 30 m/min), while the remaining roles lead players to perform from 3 to 25 m/min at sprint intensity. These values are more similar among playing positions when analyzing accelerations and decelerations (from 2 to 5 actions per minute). As a consequence of these high-intensity efforts, load indicators such as HMLD are similar among playing positions (from 23–29 to 61–72 m/min). Therefore, if the analysis of soccer players’ physical loads during the whole matched is highly useful for training individualization [40, 43], the fact of finding statistical differences among roles in the articles included in this systematic review should be considered with care, at least, with high-intensity variables such HSD, sprints, and accelerations/decelerations. The rationale for this may be the nature of WCS, that lead all players to act suddenly to try to recover a steady state and avoid the opponent’s progression towards a goal scoring opportunity. Therefore, although in general statistical differences were found among players´ positions, coaches should decide if these differences are important enough to individualize training programs aimed at developing performance for WCS depending on roles.

In brief, players´ performance is dependent on their playing positions, at least, since WD, WM, and M perform more m/min than the others. However, differences among playing positions do not seem large in those variables that explain players´ performance at high-intensity. Accordingly to most studies (~85%; 11/13 studies) [14, 15, 17, 23, 24, 26–30], it is recommended that results provided during WCS serve as the basis for future training prescription, in order to respect the specific demands imposed on players during the most demanding passages of match play.

### Worst-case scenarios depending on sex

As with men, women soccer players´ performance during the most demanding passages are time-window dependent (total distance = from 107 to 161 m/min; HSR = from 2 to 34 m/min; Acc = from 0,61 to 0,95 actions per minute; and, HMLD = from 23 to 71). Moreover, coaches should decide if the differences between playing positions (total distance = CD: 107–153 m/min, WD: 109–161 m/min, M: 116–163 m/min, WM: 109–158 m/min, and F: 107–152 m/min; HSR = CD: 3–26 m/min, WD: 5–30 m/min, M: 2–21 m/min, WM: 6–34 m/min, and F: 5–32 m/min; Acc = CD: 0,81–0,86 n/min, WD: 0,68–0,95 n/min, M: 0,62–0,87 n/min, WM: 0,67–0,95 n/min, and F: 0,61–0,88 n/min; HMLD = CD: 23–61 m/min, WD: 27–71 m/min, M: 28–66 m/min, WM: 27–67 m/min, and F: 27–67 m/min) are sufficiently important to individualize the training process by roles.

However, despite these common results between sexes, the differences between them seems to be a fact. Although women soccer players have shown greater values in some variables such as core endurance [44], the largest sex differences were evident in the explosive and intermittent endurance-related variables, with women showing lower values (large to extreme) in sprints, jumps, and intermittent endurance, and trivial to moderate differences (lower in women) in running velocity, maximal heart rate, and distance covered during incremental exercises [44]. These results lead to the hypothesis that sex differences will remain in WCS during soccer matches. In this systematic review, there were differences between men and women in distance covered (115–233 and 110–157 m/min), HSR (9–57 and 4–29 m/min), HMLD (23–72 and 26–66 m/min), and in the number of accelerations (2–4 and 0,6–0,9 n/min), respectively. These differences may arise due to anthropometric and physiological differences between the genders [45].

### Worst-case scenarios depending on contextual factors

Three studies assessed the impact of a range of contextual variables regarding the expression of WCS over match-play [12, 15, 46]. For short passages (1- to 3-min), match outcome was a significant independent factor since winners reported a higher running performance [10, 12]. Nevertheless, considering longer periods the literature showed opposed conclusions supporting [12] or contesting this result [10]. Match location impacted WCS observed in [10] whilst the study by Fereday and co-workers [12] indicated no effects in either total or high-speed distances. Finally, playing formation also contributed to WCS variations in two investigations [12, 15]. As a consequence, this indicates that previous literature on match performance in soccer [47–49] cannot be always extrapolated when analyzing the WCS in this team sport, implying a need to control for contextual constraints even in the absence of a clear consensus in some cases. In this regard, replication studies in distinct populations/locations, standards, and ages are still required to provide strong evidence, which is currently scarce according to our searches.

### Study limitations

Six main issues are recognized in the present review study: (1) Aside from the acceptable methodological scores of the majority of the papers included, there were some (14%) that did not reach high-quality levels. (2) Except in one study separating offensive and defensive game phases using a semi-automatic video tracking method [16] and another considering ball in/out of play by custom-built software coding [32], the results collected here may also lack the context in which they occurred (see for more information: [47]). This is due to the predominant use of only portable micro technologies to collect match performance (~86%) [13, 14, 17, 23–31], which alone may not allow capturing technical event occurrences like losing and regaining ball possession [48]. (3) Most importantly, whether there was any variation in players’ actual positions occupied during matches in relation to intended formation was not reported in all except two studies [27, 28], thereby indicating a need to re-examine some of the methods. One potential solution is to apply heat maps to the 2-dimensional positional data to confirm if each player indeed exerted a role predominantly in a given field location [50] (4) The information gathered is pertinent to a greater extent to men professional players, because only two literature studies determined in-game WCS in professional women [25, 28] soccer athletes. (5) Some pertinent topics consisted only of isolated/single studies (e.g. congested schedule [51], player status (such as starter/non-starter comparisons) [12], and match-to-match variability [28]), thereby making systematic recommendations unfeasible to date. (6) To end, the generalizability of current evidence to assist periodization in training is debatable [10, 28, 36]. Limitations derived either from methods adopted here or from articles considered should be carefully taken into account when interpreting systematic conclusions made, and future research is advisable to follow up such concerns.

## CONCLUSIONS

An inverse relationship existed between the length of the time window used in capturing WCS and the match-play running output in which the shortest epochs reveal the most intense in-game locomotor demands. Also, the occurrences of WCS during soccer matches at the professional level are dependent on playing position, especially analyzing performance through total distance covered. However, evidence made it possible to draw firm conclusions only in professional men players while information derived from women athletes is as yet insufficient. The inclusion of match contextual factors such as location, score status, and team formation is advisable when investigating WCS in soccer matches given their prominent influence on the expression of players’ running performance in this type of analysis. Future research should consider understanding the better methodology for measuring WCS aiming to reduce the noise, as well as determine the practical implications for real-training scenarios as to how to prescribe based on typical values.

## Funding

No other specific sources of funding were used to assist in the preparation of this article.

## Conflicts of interest/Competing interests

The authors declare that they have no conflicts of interest relevant to the content of this systematic review.

## References

[1] Paul DJ, Bradley PS, Nassis GP. Factors Affecting Match Running Performance of Elite Soccer Players: Shedding Some Light on the Complexity. Int J Sports Physiol Perform. 2015; 10(4):516–9. 25928752 10.1123/IJSPP.2015-0029

[2] Trewin J, Meylan C, Varley MC, Cronin J. The influence of situational and environmental factors on match-running in soccer: a systematic review. Sci Med Football. 2017; 1(2):183–94.

[3] Aquino R, Vieira LHP, Carling C, Martins GHM, Alves IS, Puggina EF. Effects of competitive standard, team formation and playing position on match running performance of Brazilian professional soccer players. Int J Perform Anal Sport. 2017; 17(5):695–705.

[4] Trewin J, Meylan C, Varley MC, Cronin J, Ling D. Effect of Match Factors on the Running Performance of Elite Female Soccer Players. 2018; 32(7):2002–9. 10.1519/JSC.0000000000002584 29570576

[5] Buchheit M, Simpson BM, Hader K, Lacome M. Occurrences of near-to-maximal speed-running bouts in elite soccer: insights for training prescription and injury mitigation. Sci Med Football. 2020 Aug 5; 1–6. 10.1080/24733938.2020.1802058 35077328

[6] Miguel M, Oliveira R, Loureiro N, García-Rubio J, Ibáñez SJ. Load Measures in Training/Match Monitoring in Soccer: A Systematic Review. Int J Environ Res Public Health. 2021; 18(5):2721. 33800275 10.3390/ijerph18052721 PMC7967450

[7] Oliva-Lozano JM, Gómez-Carmona CD, Pino-Ortega J, Rodríguez-Pérez MA. Match and Training High Intensity Activity-Demands Profile during a Competitive Mesocycle in Youth Elite Soccer Players. J Hum Kinet. 2020; 75:195–205. 33312307 10.2478/hukin-2020-0050 PMC7706676

[8] Schimpchen J, Gopaladesikan S, Meyer T. The intermittent nature of player physical output in professional football matches: An analysis of sequences of peak intensity and associated fatigue responses. Eur J Sport Sci. 2020 Jun 15; 1–10. 10.1080/17461391.2020.1776400 32466742

[9] Oliva-Lozano JM, Muyor JM, Fortes V, McLaren SJ. Decomposing the variability of match physical performance in professional soccer: Implications for monitoring individuals. Eur J Sport Sci. 2020 Nov 22; 1–9. 10.1080/17461391.2020.1842513 33100192

[10] Oliva-Lozano JM, Rojas-Valverde D, Gómez-Carmona CD, Fortes V, Pino-Ortega J. Worst case scenario match analysis and contextual variables in professional soccer players: a longitudinal study. Biol Sport. 2020; 37(4):429–36. 33343077 10.5114/biolsport.2020.97067 PMC7725043

[11] Doncaster G, Page R, White P, Svenson R, Twist C. Analysis of Physical Demands During Youth Soccer Match-Play: Considerations of Sampling Method and Epoch Length. Res Q Exerc Sport. 2020; 91(2):326–34. 31774386 10.1080/02701367.2019.1669766

[12] Fereday K, Hills SP, Russell M, Smith J, Cunningham DJ, Shearer D, McNarry M, Kilduff LP. A comparison of rolling averages versus discrete time epochs for assessing the worst-case scenario locomotor demands of professional soccer match-play. J Sci Med Sport. 2020; 23(8):764–9. 31937507 10.1016/j.jsams.2020.01.002

[13] Oliva-Lozano JM, Fortes V, M. Muyor J . The first, second, and third most demanding passages of play in professional soccer: a longitudinal study. Biol Sport. 2021; 38(2):165–74. 34079161 10.5114/biolsport.2020.97674 PMC8139346

[14] Harkness-Armstrong A, Till K, Datson N, Emmonds S. Whole and peak physical characteristics of elite youth female soccer match-play. J Sports Sci. 2021; 1–10. 10.1080/02640414.2020.1868669 33377422

[15] Riboli A, Semeria M, Coratella G, Esposito F. Effect of formation, ball in play and ball possession on peak demands in elite soccer. Biol Sport. 2021; 38(2):195–205. 34079164 10.5114/biolsport.2020.98450 PMC8139352

[16] Tomazoli G, Marques JB, Farooq A, Silva JR. Estimating Postmatch Fatigue in Soccer: The Effect of Individualization of Speed Thresholds on Perceived Recovery. Int J Sports Physiol Perform. 2020; 15(9):1216–22. 10.1123/ijspp.2019-0399 32422598

[17] Whitehead S, Till K, Weaving D, Jones B. The Use of Microtechnology to Quantify the Peak Match Demands of the Football Codes: A Systematic Review. Sports Med. 2018; 48(11):2549–75. 30088218 10.1007/s40279-018-0965-6 PMC6182461

[18] Rico-González M, Pino-Ortega J, Clemente F, Los Arcos A. Guidelines for performing systematic reviews in sports science. Biol Sport. 2022; 39(3). 10.5114/biolsport.2022.106386 PMC8919872 35309539

[19] Group CCCR. Data Extraction Template for Included Studies. 2016.

[20] O’Reilly M, Caulfield B, Ward T, Johnston W, Doherty C. Wearable Inertial Sensor Systems for Lower Limb Exercise Detection and Evaluation: A Systematic Review. Sports Med. 2018; 48(5):1221–46. 29476427 10.1007/s40279-018-0878-4

[21] Baptista I, Johansen D, Figueiredo P, Rebelo A, Pettersen SA. Positional Differences in Peak- and Accumulated-Training Load Relative to Match Load in Elite Football. Sports. 2019; 8(1):1. 10.3390/sports8010001 PMC7023441 31877942

[22] Casamichana D, Castellano J, Diaz AG, Gabbett TJ, Martin-Garcia A. The most demanding passages of play in football competition:a comparison between halves. Biol Sport. 2019; 36(3):233–40. 31624417 10.5114/biolsport.2019.86005 PMC6786330

[23] Martín-García A, Casamichana D, Díaz AG, Cos F. Positional Differences in the Most Demanding Passages of Play in Football Competition. J Sports Sci Med. 2018; 17:563–70. 30479524 PMC6243617

[24] Martin-Garcia A, Castellano J, Diaz AG, Cos F, Casamichana D. Positional demands for various-sided games with goalkeepersaccording to the most demanding passages of match playin football. Biol Sport. 2019; 36(2):171–80. 31223195 10.5114/biolsport.2019.83507 PMC6561222

[25] Muñiz-González J, Giráldez-Costas V, González-García J, Romero-Moraleda B, Campos-Vázquez MÁ. Diferencias posicionales en las fases de máxima exigencia condicional en fútbol femenino. [Positional differences in the most demanding conditional phases in female football competition]. RYCIDE. 2020; 16(60):199–213.

[26] Oliva-Lozano JM, Gómez-Carmona CD, Rojas-Valverde D, Fortes V, Pino-Ortega J. Effect of training day, match, and length of the microcycle on the worst-case scenarios in professional soccer players. Res Sports Med. 2021 Mar 4; 1–14. 10.1080/15438627.2021.1895786 33657955

[27] Riboli A, Esposito F, Coratella G. The distribution of match activities relative to the maximal intensities in elite soccer players: implications for practice. Res Sports Med. 2021; 1–12. 10.1080/15438627.2021.1895788 33657944

[28] Trewin J, Meylan C, Varley MC, Cronin J. The match-to-match variation of match-running in elite female soccer. J Sci Med Sport. 2018; 21(2):196–201. 28595867 10.1016/j.jsams.2017.05.009

[29] McGarry T, Anderson DI, Wallace SA, Hughes MD, Franks IM. Sport competition as a dynamical self-organizing system. J Sports Sci. 2002; 20(10):771–81. 12363294 10.1080/026404102320675620

[30] Clemente F, Sarmento H. The effects of small-sided soccer games on technical actions and skills: A systematic review. Hum Mov. 2020; 21(3):100–19.

[31] Schmidt, R. C., O´ Brien, B., & Sysko, R. Self organization of between person cooperative tasks and possible applications to sport. Int J Sport Psychol. 1999; 30:558–79.

[32] Del Coso J, Brito de Souza D, Moreno-Perez V, Buldú JM, Nevado F, Resta R, López-Del Campo R. Influence of Players’ Maximum Running Speed on the Team’s Ranking Position at the End of the Spanish LaLiga. Int J Environ Res Public Health. 2020; 17(23):8815. 10.3390/ijerph17238815 PMC7729782 33261014

[33] Di Salvo V, Gregson W, Atkinson G, Tordoff P, Drust B. Analysis of High Intensity Activity in Premier League Soccer. Int J Sports Med. 2009; 30(03):205–12. 19214939 10.1055/s-0028-1105950

[34] Pino-Ortega J, Rojas-Valverde D, Gómez-Carmona CD, Rico-González M. Training Design, Performance Analysis and Talent Identification—A Systematic Review about the Most Relevant Variables through the Principal Component Analysis in Soccer, Basketball and Rugby. Int J Environ Res Public Health. 2021; 18. 33807971 10.3390/ijerph18052642 PMC7967544

[35] Casamichana D, Castellano J, Gómez Díaz A, Martín-García A. Looking for Complementary Intensity Variables in Different Training Games in Football: J Strength Cond Res. 2019 Mar; 1. 30844980 10.1519/JSC.0000000000003025

[36] Novak AR, Impellizzeri FM, Trivedi A, Coutts AJ, McCall A. Analysis of the worst-case scenarios in an elite football team: Towards a better understanding and application. J Sports Sci. 2021 Apr 10; 1–10. 10.1080/02640414.2021.1902138 33840362

[37] Castellano J, Pic M. Identification and Preference of Game Styles in LaLiga Associated with Match Outcomes. Int J Environ Res Public Health. 2019; 16(24):5090. 10.3390/ijerph16245090 PMC6950299 31847147

[38] Clemente F, Silva R, Ramirez-Campillo R, Afonso J, Mendes B, Chen Y-S. Accelerometry-based variables in professional soccer players: Comparisons between periods of the season and playing positions. Biol Sport. 2020; 37(4):389–403. 33343073 10.5114/biolsport.2020.96852 PMC7725036

[39] Borghi S, Colombo D, La Torre A, Banfi G, Bonato M, Vitale JA. Differences in GPS variables according to playing formations and playing positions in U19 male soccer players. Res Sports Med. 2020 Sep 3; 1–15. 10.1080/15438627.2020.1815201 32880481

[40] Andrzejewski M, Chmura J, Pluta B, Kasprzak A. Analysis of Motor Activities of Professional Soccer Players. J Strength Cond Res. 2012; 26(6):1481–8. 22614138 10.1519/JSC.0b013e318231ab4c

[41] Di Salvo V, Baron R, Tschan H, Calderon Montero F, Bachl N, Pigozzi F. Performance Characteristics According to Playing Position in Elite Soccer. Int J Sports Med. 2007; 28(3):222–7. 17024626 10.1055/s-2006-924294

[42] Altavilla G, Riela L, Tore APD, Raiola G. The physical effort required from professional football players in different playing positions. J Physic Edu Sport. 2017; 6.

[43] Wass J, Mernagh D, Pollard B, Stewart P, Fox W, Parmar N, Jones B, Kilduff L, Turner AN. A comparison of match demands using ball-in-play vs. whole match data in elite male youth soccer players. Sci Med Football. 2020; 4(2):142–7.

[44] Cardoso de Araújo M, Baumgart C, Jansen CT, Freiwald J, Hoppe MW. Sex Differences in Physical Capacities of German Bundesliga Soccer Players. J Strength Cond Res. 2020; 34(8):2329–37. 29927885 10.1519/JSC.0000000000002662

[45] Tenga A, Zubillaga A, Caro O, Fradua L. Explorative Study on Patterns of Game Structure in Male and Female Matches from Elite Spanish Soccer. Int J Perform Anal Sport. 2015; 15(1):411–23.

[46] Oliva-Lozano JM, Rojas-Valverde D, Gómez-Carmona CD, Fortes V, Pino-Ortega J. Impact of contextual variables on the representative external load profile of Spanish professional soccer match-play: A full season study. Eur J Sport Sci. 2020 May 12; 1–10. 10.1080/17461391.2020.1751305 32233969

[47] Palucci Vieira LH, Carling C, Barbieri FA, Aquino R, Santiago PRP. Match Running Performance in Young Soccer Players: A Systematic Review. Sports Med. 2019; 49(2):289–318. 30671900 10.1007/s40279-018-01048-8

[48] Carling C, Bloomfield J, Nelsen L, Reilly T. The role of motion analysis in elite soccer: contemporary performance measurement techniques and work rate data. Sports Med. 2008; 38(10):839–62. 18803436 10.2165/00007256-200838100-00004

[49] Lago-Peñas C. The role of situational variables in analysing physical performance in soccer. J Hum Kinet. 2012; 35:89–95. 23487326 10.2478/v10078-012-0082-9 PMC3588697

[50] Moura FA, Santana JE, Vieira NA, Santiago PRP, Cunha SA. Analysis of Soccer Players’ Positional Variability During the 2012 UEFA European Championship: A Case Study. J Hum Kinet. 2015; 47:225–36. 26557206 10.1515/hukin-2015-0078 PMC4633258

[51] Castellano J, Martin-Garcia A, Casamichana D. Most running demand passages of match play in youth soccer congestion period. Biol Sport. 2020; 37(4):367–73. 33343070 10.5114/biolsport.2020.96853 PMC7725039

